# The Acute Effects of Grape Polyphenols Supplementation on Endothelial Function in Adults: Meta-Analyses of Controlled Trials

**DOI:** 10.1371/journal.pone.0069818

**Published:** 2013-07-24

**Authors:** Shao-Hua Li, Hong-Bo Tian, Hong-Jin Zhao, Liang-Hua Chen, Lian-Qun Cui

**Affiliations:** Department of Cardiology, Provincial Hospital Affiliated to Shandong University, Jinan, China; University of Tor Vergata, Italy

## Abstract

**Background:**

The acute effects of grape polyphenols on endothelial function in adults are inconsistent. Here, we performed meta-analyses to determine these acute effects as measured by flow-mediated dilation (FMD).

**Methods:**

Trials were searched in PubMed, Embase and the Cochrane Library database. Summary estimates of weighted mean differences (WMDs) and 95% CIs were obtained by using random-effects models. Meta-regression and subgroup analyses were performed to identify the source of heterogeneity. The protocol details of our meta-analysis have been submitted to the PROSPERO register and our registration number is CRD42013004157.

**Results:**

Nine studies were included in the present meta-analyses. The results showed that the FMD level was significantly increased in the initial 120 min after intake of grape polyphenols as compared with controls. Meta-regression and subgroup analyses were performed and showed that a health status was the main effect modifier of the significant heterogeneity. Subgroups indicated that intake of grape polyphenols could significantly increase FMD in healthy subjects, and the increased FMD appeared to be more obviously in subjects with high cardiovascular risk factors. Moreover, the peak effect of grape polyphenols on FMD in healthy subjects was found 30 min after ingestion, which was different from the effect in subjects with high cardiovascular risk factors, in whom the peak effect was found 60 min after ingestion.

**Conclusions:**

Endothelial function can be significantly improved in healthy adults in the initial 2 h after intake of grape polyphenols. The acute effect of grape polyphenols on endothelial function may be more significant but the peak effect is delayed in subjects with a smoking history or coronary heart disease as compared with the healthy subjects.

## Introduction

Atherosclerosis is the primary pathophysiological basis for most cardiovascular diseases [1], and endothelial dysfunction has been considered an early feature in the progression of atherosclerosis and an independent predictor of poor prognosis in many cardiovascular diseases [2], [3]. Improvement of endothelial function in daily life has been recommended in recent years [4] and grape polyphenols, which are mainly extracted from red grape, have been found to have cardioprotective effects and improve endothelial function in many experimental studies [5]–[8].

Extracted grape polyphenols contain high concentrations of epicatechin, catechin, quercetin, gallic acid and resveratrol as well as other compounds [9]. *In vitro* studies have indicated that grape polyphenols can enhance the endothelial nitric oxide synthase and increase the production of nitric oxide in endothelial cells [6], [7]. Administration of grape polyphenols could significantly increase the nitric oxide level in endothelial cells and improve endothelial function in hypercholesterolemic rabbits [10]. The intriguing results *in vitro* and animal studies encouraged people to investigate the effects of grape polyphenols on endothelial function in adults. Due to the rapidly metabolized feature of polyphenols [11], most clinical trials in adults have focused on the acute effects of grape polyphenols on endothelial function [12]–[38]. However, the results of these studies were not consistent, and the sample sizes were relatively small, resulting in inconsistent conclusions regarding the acute effects of grape polyphenols.

Hooper *et al.* published 4 trials (before 2007) and conducted a meta-analysis [4] investigating the acute effects of grape polyphenols on endothelial function. However, in the meta-analysis, the acute effects of grape polyphenols on endothelial function were not explored at fixed times (e.g., 30 min, 60 min, 120 min after ingestion) and many new trials have been reported since 2007. Therefore, new meta-analyses are needed to clarify the acute effects of grape polyphenols on endothelial function in adults.

Flow-mediated vasodilation (FMD) of the brachial artery, a noninvasive ultrasound method to assess endothelial function [39], [40], has been carried out by most of the trials investigating the acute effects of grape polyphenols. In the present study, we identified all published and controlled trials of grape polyphenols and performed meta-analyses to evaluate the acute effects of grape polyphenols on FMD in adults.

## Materials and Methods

The protocol details of our present meta-analyses have been submitted to the PROSPERO register and this record has been published on the database at http://www.crd.york.ac.uk/prospero/ **.** Our registration number is *CRD42013004157*. Meanwhile, the protocol details and the PRISMA checklist have also been provided in Table S1 and Table S2.

### Literature Search

The present meta-analyses were conducted according to the Preferred Reporting Items for Systematic reviews and Meta-Analyses (PRISMA) guidelines [41]. We systematically searched PubMed (from 1950 to Jan, 2013), EMBASE (from 1966 to Jan, 2013), and the Cochrane Library for published reports by using the query “(grape) OR (polyphenol) OR (red wine)” paired with “(endothelial) OR (endothelium)”. Reference lists of articles were also analyzed using a manual approach.

### Study Selection

Studies were chosen for analysis if they met the following criteria: (i) the article was published in English; (ii) studies were controlled trials in adults; (iii) the endothelial function was evaluated by the flow-mediated vasodilation method; (iv) FMD was measured after fasting and at fixed times (e.g., 30 min, 60 min, 120 min, 180 min, etc.) after the intake of the grape polyphenols; (v) the values of FMD were reported at the start and end of the intervention.

### Data Extraction and Quality Assessment

The search, data extraction, and quality assessment were completed independently by two reviewers according to the inclusion criteria. Any discrepancies between the two reviewers were resolved through discussion until a consensus was reached. The extracted data included the study characteristics, population information, and the baseline and final FMD values. If red wine and de-alcoholized red wine were both used as the supplementation of grape polyphenols, both the alcoholized and de-alcoholized data were extracted and separated into two independent trials (alcoholized trial and de-alcoholized trial).

The quality of the studies was judged by concealment of treatment allocation, quality of randomization, blinding, reporting of withdrawals, and generation of random numbers. Trials were scored one point for each area addressed, with a possible score of 0 to 5 (highest level of quality) [42].

### Statistical Analysis

The primary outcome was the percentage change in FMD between baseline and final levels due to grape polyphenols supplementation. If the percentage change in FMD was not reported in the study, we calculated it according to the Cochrane Handbook for Systematic Review and Follman D’s theory for overview of clinical trials with continuous variables [43]. We assumed equal variance among trials and between intervention and controls. Weighted mean differences and 95% confidence intervals (CIs) were calculated for net changes in FMD values [44]. Statistic heterogeneity of treatment effects between studies was formally tested with Cochran’s test (*P*<0. 1). The *I* ^2^ statistic was also examined, and we considered an *I* ^2^ value >50% to indicate significant heterogeneity between the trials [45]. Potential heterogeneity in estimates of treatment effect was explored by univariate meta-regression. Furthermore, subgroup analyses were also performed to identify the possible sources of heterogeneity by comparing summary results obtained from subsets of studies grouped by age, health status of the subjects, the source and dose of grape polyphenols, and baseline FMD level.

Publication bias was assessed with the Egger regression test and funnel plots [46]. Meta-analyses and statistical analyses were performed with Stata software (version 10.0; Stata Corporation, College Station, TX, USA) and REVMAN software (version 5.0; Cochrane Collaboration, Oxford, UK).

## Results

### Search Results

**Figure 1:**
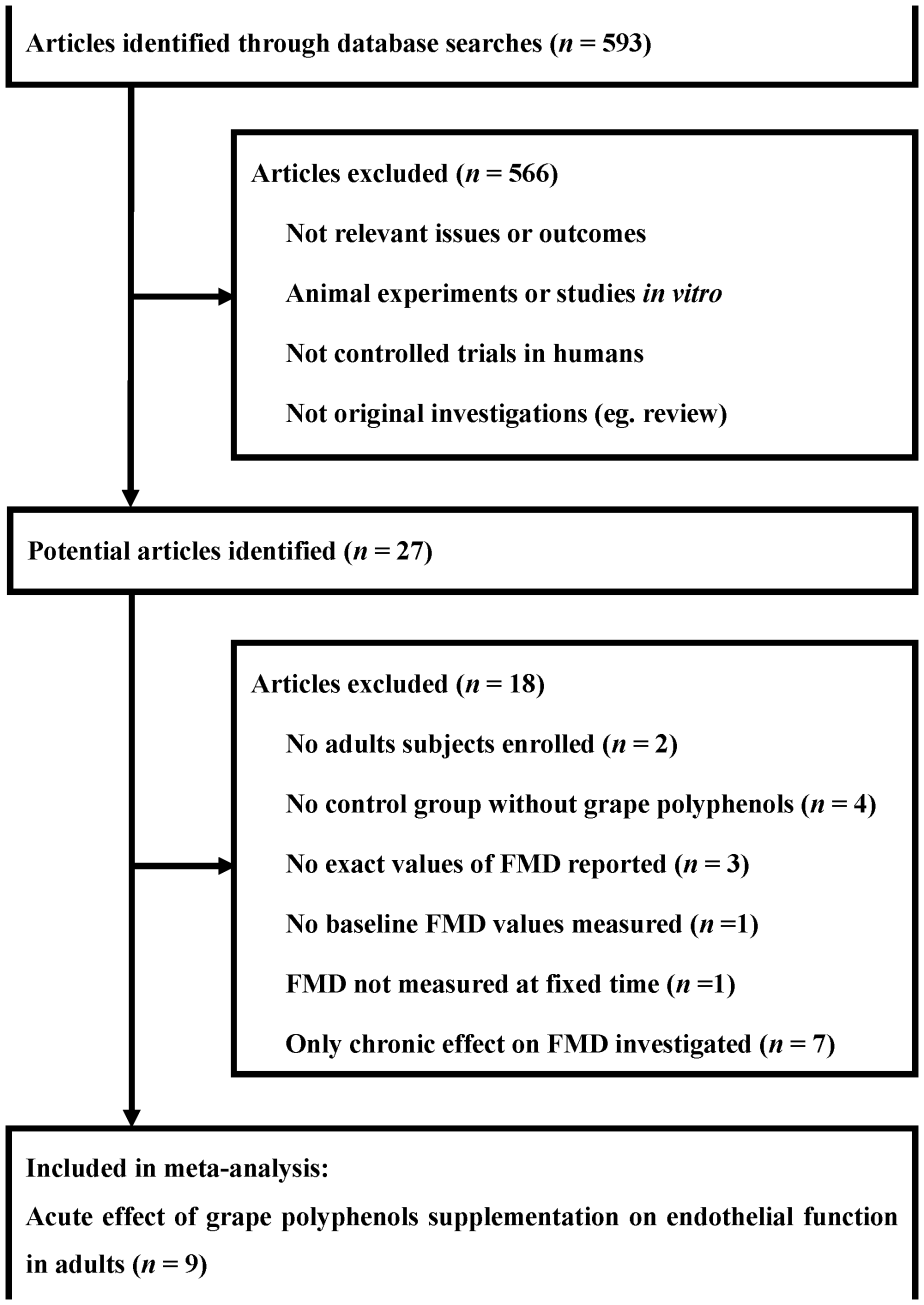
Identification process for eligible studies. FMD, flow-mediated dilation.

The identification process of eligible studies is shown in Figure 1. A total of 593 articles were identified in a combined search of the PubMed, Embase, and Cochrane Library databases. Of the 593 articles, 566 were excluded because they were studied in animals or *in vitro*, or because the objectives were not related to the present meta-analyses. Therefore, 27 potentially relevant articles were selected for full text evaluation [12]–[38]. Of these, we included 9 eligible controlled studies [12]–[20] in our present meta-analyses. The remaining 18 articles were excluded for the following reasons: the enrolled subjects were adolescents but not adults [21], [22]; there were no control groups in the studies [23], [24], or all the groups designed in the studies used grape polyphenols and no blank control groups were included (intervention without grape polyphenols) [25], [26]; the baseline FMD was not measured in one trial [27]; the exact values of FMD were not reported in three trials [28]–[30]; the measurements of FMD were based on the peak blood alcohol concentration, but not at fixed times (for example, 30 min, 60 min, 120 min after ingestion) in one study [31]; there were 7 trials investigating the chronic but not acute effects of grape polyphenols on the endothelial function [32]–[38].

### Study Characteristics

Nine studies were included in the present meta-analyses. The characteristics of the included trials are shown in Table 1. All nine studies were controlled trials. The average age of the subjects ranged from 22 to 61 years. Of the nine trials, seven trials included healthy adults [12]–[14], [16], [17], [19], [20], and the other two enrolled smokers [18] or patients with coronary heart disease [15]. Three studies reported that the mean basal total cholesterol of the enrolled subjects was less than 6.0 mmol/L [14], [18], [19], whereas the mean basal total cholesterol level in the other three studies varied from 4.3 to 5.45 mmol/L [12], [15], [17]. The baseline triglyceride concentration was only reported in three trials [12], [15], [17], and ranged from 1.0 mmol/L to 1.80 mmol/L.

**Table 1:** Characteristics of the included trials investigating the acute effects of grape polyphenols.

Author	Year	Design	Number of subjects	Mean age of subjects (year)	Health status of subjects	Source of grape polyphenols	Dose of grape polyphenols	Mean basal total cholesterol	Mean basal triglyceride	Side effects reported
Djousse et al	1999	CO	13	32	Healthy	Red wine	∼ 600 mg	4.3 mmol/L	1.0 mmol/L	No
Hashimoto et al *De-alcoholized* 1	2001	R PC CO	11	34	Healthy	Red wine without alcohol	1000 mg	Not reported	Not reported	No
Hashimoto et al *Alcoholized* 1	2001	R PC CO	11	34	Healthy	Red wine	1000 mg	Not reported	Not reported	No
Papamichael et al *De-alcoholized* 1	2004	DB CO	16	28.9	Healthy	Red wine without alcohol	∼ 650 mg	<6.0 mmol/L	Not reported	No
Papamichael et al *Alcoholized* 1	2004	DB CO	16	28.9	Healthy	Red wine	∼ 650 mg	<6.0 mmol/L	Not reported	No
Lekakis et al	2005	R PC	30	61	Coronary heart disease	Red grape polyphenol extract	600 mg	5.45 mmol/L	1.80 mmol/L	No
Boban et al *De-alcoholized* 1	2006	R PC CO	9	33	Healthy	Red wine without alcohol	∼660 mg	Not reported	Not reported	No
Boban et al *Alcoholized* 1	2006	R PC CO	9	33	Healthy	Red wine	∼660 mg	Not reported	Not reported	No
Karatzi et al *De-alcoholized* 1	2007	DB PC CO	20	29	Smoking	Red wine without alcohol	∼ 650 mg	<6.0 mmol/L	Not reported	No
Karatzi et al *Alcoholized* 1	2007	DB PC CO	20	29	Smoking	Red wine	∼ 650 mg	<6.0 mmol/L	Not reported	No
Hijmering et al	2007	R	20	35	Healthy	Red wine	∼ 850 mg	4.7 mmol/L	1.37 mmol/L	No
Karatzi et al *Olive* 2	2008	R CO	15	29.5	Healthy	Red wine	∼ 660 mg	<6.0 mmol/L	Not reported	No
Karatzi et al *Green Olive* 2	2008	R CO	15	29.5	Healthy	Red wine	∼ 660 mg	<6.0 mmol/L	Not reported	No
Hampton et al *De-alcoholized* 1	2010	R PC CO	10	22	Healthy	Organic red grape juice	∼ 1200 mg	Not reported	Not reported	No
Hampton et al *Alcoholized* 1	2010	R PC CO	10	22	Healthy	Organic red grape juice and alcohol	∼ 1200 mg	Not reported	Not reported	No

R, randomized; DB, double-blind; PC, placebo-controlled; CO, crossover; FMD, flow-mediated dilation.

1 Study designed alcoholized group and de-alcoholized group to investigate the effects of grape polyphenols on FMD; therefore, we separated the study into 2 trials: alcoholized trial and de-alcoholized trial.

2 Karatzi’s study designed red wine+olive group and red wine+green olive group to investigate the effect of grape polyphenols on FMD respectively, so we separated Karatzi’s study into 2 trials: olive trial and green olive trial.

There were five trials [13], [14], [16], [18], [20] which designed an alcoholized and a de-alcoholized group to investigate the effect of grape polyphenols on FMD. Therefore, the alcoholized and de-alcoholized groups were separated into two independent trials in the present meta-analyses. A trial by Karatzi [19] detected the acute effects of grape polyphenols by using red wine with olive oil or green olive oil; therefore, we also separated them into two independent trials (red wine+olive trial and red wine+green olive trial). The baseline and final FMD levels of the included trials are shown in Table 2 and Table 3.

**Table 2:** The baseline and final FMD levels of the included trials (30 min and 60 min).

		30 min		60 min	
Authors	Year	Grape polyphenols group Baseline/Final FMD (%)	Control group Baseline/Final FMD (%)	Grape polyphenols group Baseline/Final FMD (%)	Control group Baseline/Final FMD (%)
Djousse et al	1999	Not reported	Not reported	Not reported	Not reported
Hashimoto et al *De-alcoholized*	2001	7.4±2.95/12.0±5.80	7.0±2.25/7.8±3.56	Not reported	Not reported
Hashimoto et al *Alcoholized*	2001	7.2±2.10/7.1±4.83	7.0±2.25/7.8±3.56	Not reported	Not reported
Papamichael et al *De-alcoholized*	2004	5.80±2.10/6.08±3.60	6.52±2.40/2.27±2.00	5.8±2.10/5.66±2.50	6.52±2.40/4.10±2.60
Papamichael et al *Alcoholized*	2004	5.79±2.10/4.05±1.80	6.52±2.40/2.27±2.00	5.79±2.10/4.87±2.40	6.52±2.40/4.10±2.60
Lekakis et al	2005	2.60±1.50/3.73±2.10	2.75±1.85/2.62±1.65	2.60±1.50/4.52±1.34	2.75±1.85/2.64±1.80
Boban et al *De-alcoholized*	2006	Not reported	Not reported	8.80±2.70/7.46±2.07	8.49±1.83/7.48±1.65
Boban et al *Alcoholized*	2006	Not reported	Not reported	7.11±1.82/8.83±1.91	8.49±1.83/7.48±1.65
Karatzi et al *De-alcoholized*	2007	4.94±3.58/6.93±5.37	5.65±3.44/2.07±3.13	4.94±3.58/6.24±3.13	5.65±3.44/2.25±2.32
Karatzi et al *Alcoholized*	2007	5.11±2.01/4.09±2.25	5.65±3.44/2.07±3.13	5.11±2.01/6.14±1.74	5.65±3.44/2.25±2.32
Hijmering et al	2007	Not reported	Not reported	Not reported	Not reported
Karatzi et al *Olive*	2008	Not reported	Not reported	6.60±3.10/6.90±4.65	7.20±2.71/6.50±2.71
Karatzi et al *Green Olive*	2008	Not reported	Not reported	5.90±2.32/10.5±3.48	7.50±4.26/6.80±3.10
Hampton et al *De-alcoholized*	2010	5.80±0.60/7.55±4.80	5.40±1.30/5.50±2.40	5.80±0.60/6.10±2.20	5.40±1.30/4.40±0.80
Hampton et al *Alcoholized*	2010	5.80±1.10/8.40±2.00	5.40±1.30/5.50±2.40	5.80±1.10/6.00±2.30	5.40±1.30/4.40±0.80

Data expressed as the means ± SD.

FMD, flow-mediated dilation.

**Table 3:** The baseline and final FMD levels of the included trials (120 min and 180 min).

		120 min		180 min	
Authors	Year	Grape polyphenols group Baseline/Final FMD (%)	Control group Baseline/Final FMD (%)	Grape polyphenols group Baseline/Final FMD (%)	Control group Baseline/Final FMD (%)
Djousse et al	1999	5.57±1.43/6.98±2.51	6.04±1.66/6.37±1.61	Not reported	Not reported
Hashimoto et al *De-alcoholized*	2001	7.4±2.95/9.0±3.32	7.0±2.25/7.6±3.56	Not reported	Not reported
Hashimoto et al *Alcoholized*	2001	7.2±2.10/8.8±2.32	7.0±2.25/7.6±3.56	Not reported	Not reported
Papamichael et al *De-alcoholized*	2004	Not reported	Not reported	Not reported	Not reported
Papamichael et al *Alcoholized*	2004	Not reported	Not reported	Not reported	Not reported
Lekakis et al	2005	2.60±1.50/4.10±2.60	2.75±1.85/2.73±1.80	Not reported	Not reported
Boban et al *De-alcoholized*	2006	Not reported	Not reported	Not reported	Not reported
Boban et al *Alcoholized*	2006	Not reported	Not reported	Not reported	Not reported
Karatzi et al *De-alcoholized*	2007	Not reported	Not reported	Not reported	Not reported
Karatzi et al *Alcoholized*	2007	Not reported	Not reported	Not reported	Not reported
Hijmering et al	2007	Not reported	Not reported	8.60±1.80/1.20±2.60	7.30±4.80/1.20±3.30
Karatzi et al *Olive*	2008	6.60±3.10/7.80±3.10	7.20±2.71/7.30±2.32	6.60±3.10/6.80±3.10	7.20±2.72/6.60±3.10
Karatzi et al *Green Olive*	2008	5.90±2.32/9.0±3.10	7.50±4.26/6.90±2.71	5.90±2.32/7.80±3.87	7.50±4.26/6.70±3.48
Hampton et al *De-alcoholized*	2010	Not reported	Not reported	Not reported	Not reported
Hampton et al *Alcoholized*	2010	Not reported	Not reported	Not reported	Not reported

Data expressed as the means ± SD.

FMD, flow-mediated dilation.

The quality score of the nine studies ranged from 1 to 4. Six were randomized, controlled studies [13], [15]–[17], [19], [20], and two were double-blinded studies [14], [18]. Five of the nine studies reported the details of withdrawals [12]–14,18,20, whereas the other four studies did not address this issue [15]–[17], [19].

### Acute Effects of Grape Polyphenols on FMD 30 min after the Intervention

**Figure 2:**
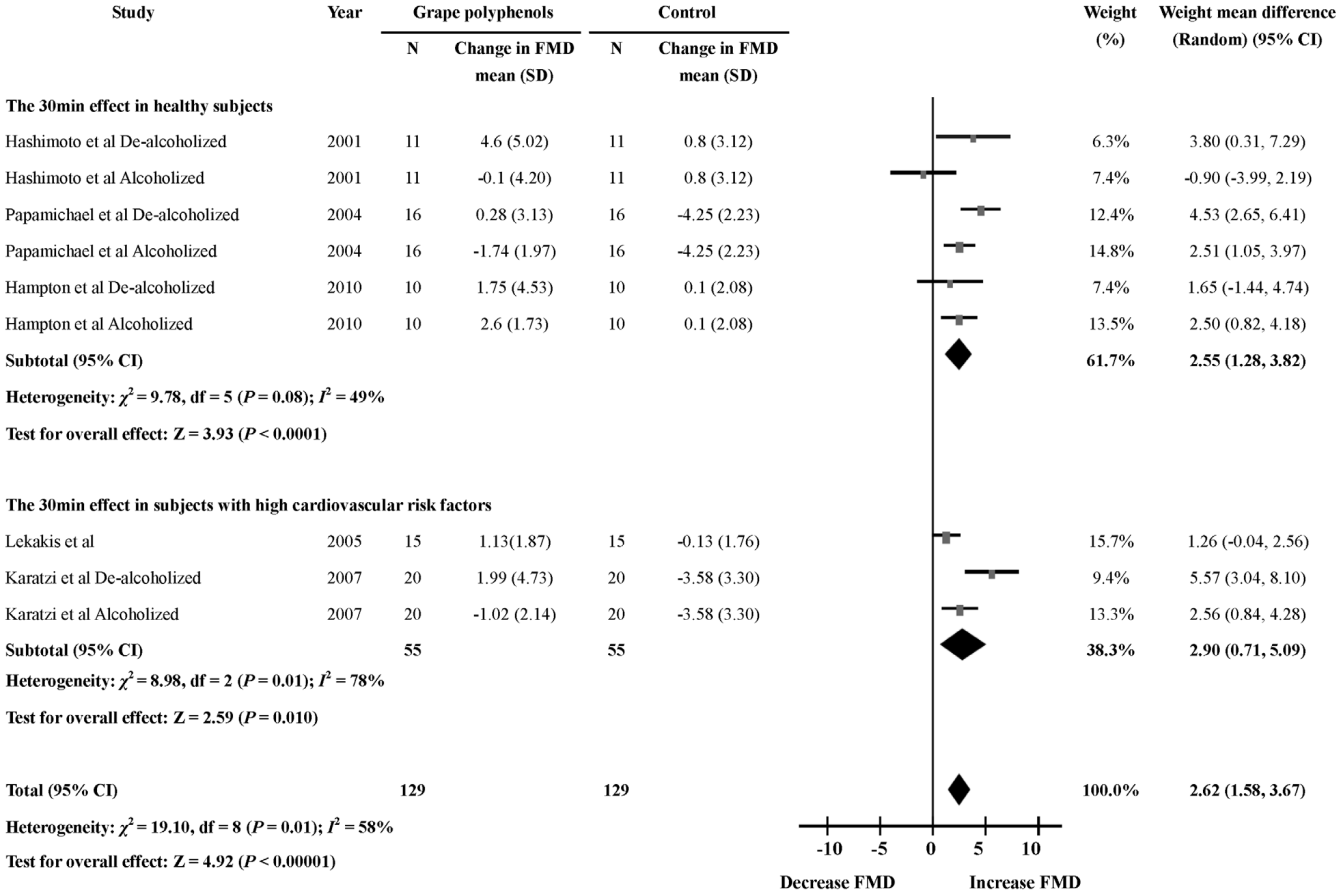
Meta-analysis of the 30 min effects of grape polyphenols on flow-mediated dilation (FMD) compared with control subjects. The sizes of the data markers indicate the weight of each study in the analysis. The subgroups were differentiated by health status of the subjects.

In the included nine studies, five [13]–[15], [18], [20] reported the effects of grape polyphenols on FMD 30 min after intervention. The meta-analysis showed that the percentage change in FMD level 30 min after the intervention was significantly higher in the grape polyphenols-supplemented subjects than in control subjects (9 comparisons; WMD:2.62%; 95% CI: 1.58, 3.67; *P*<0.00001) (Figure 2). Significant heterogeneity was found (heterogeneity *I* ^2^ = 58%, *P* = 0.01). Meta-regression was performed to detect the sources of heterogeneity, and the results indicated that health status might be the main effect modifier (*P*<0.05). The dose of grape polyphenols, the intervention with alcohol or without alcohol, the baseline FMD level and the average age of the participants were not effect modifiers.

To clarify the heterogeneity, subgroup analysis based on the health status of enrolled subjects was performed and the results showed that the grape polyphenols could significantly increase FMD level 30 min after the intervention in the healthy subgroup (6 comparisons; WMD:2.55%; 95% CI: 1.28, 3.82; *P*<0.0001). In the subgroup with high cardiovascular risk factors, intake of grape polyphenols appeared to be a more significant influence on FMD when compared to the healthy subgroup 30 min after the intervention (3 comparisons; WMD: 2.90%; 95% CI: 0.71, 5.09; *P* = 0.010). Furthermore, the heterogeneity of the effect has been largely explained by the health status, and there was no significant heterogeneity in the healthy subgroup (heterogeneity *I* ^2^ = 49%, *P* = 0.08) (Figure 2, Table S3).

### Acute Effects of Grape Polyphenols on FMD 60 min after the Intervention

**Figure 3:**
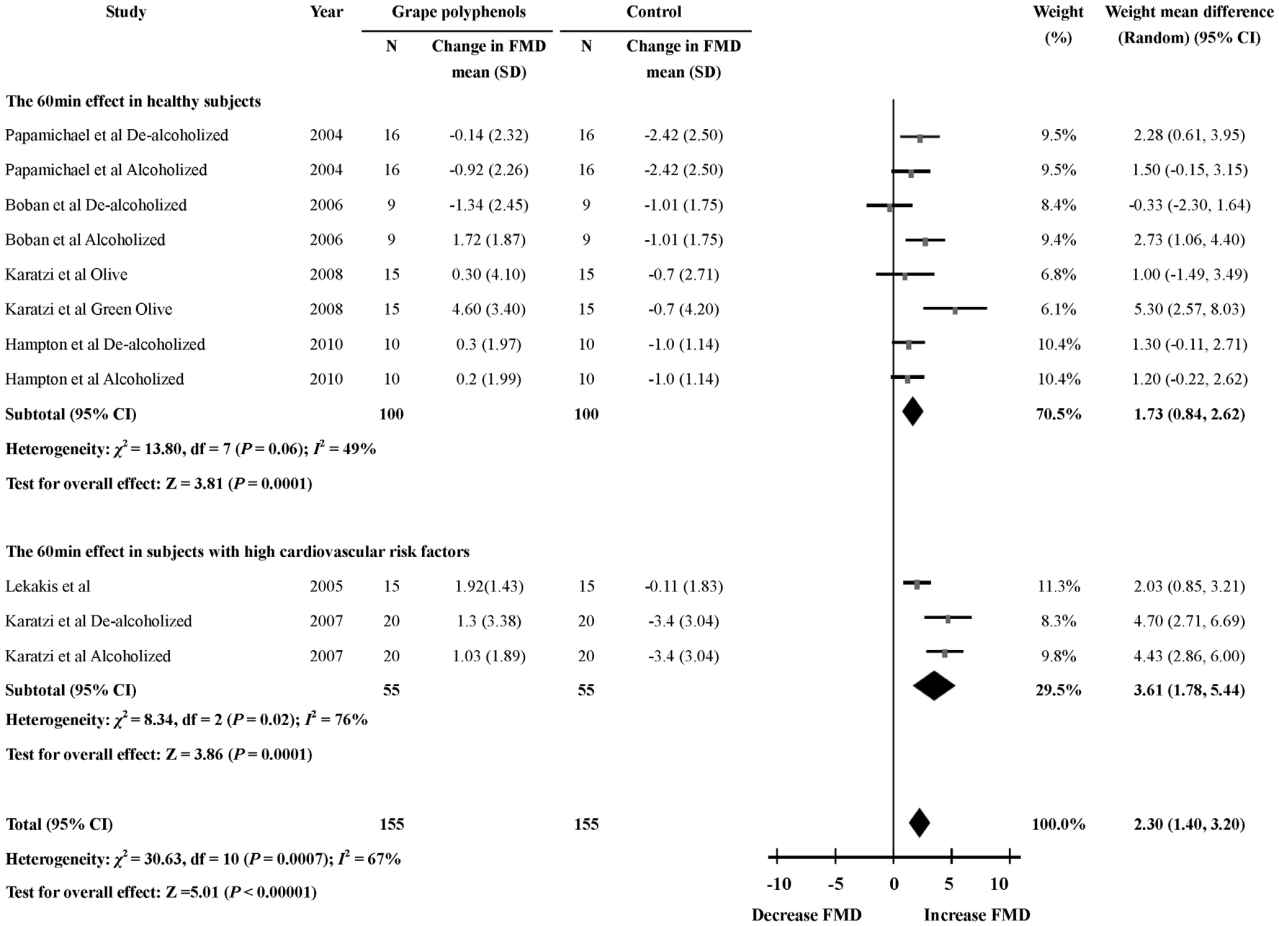
Meta-analysis of the 60 min effect of grape polyphenols on flow-mediated dilation (FMD) compared with controls. The sizes of the data markers indicate the weight of each study in the analysis. The subgroups were differentiated by health status of the subjects.

Six studies [14]–[16], [18]–[20] reported the effects of grape polyphenols on FMD 60 min after intervention. The percentage change in FMD 60 min after the intervention was significantly higher in the grape polyphenols group than the control group (11 comparisons; WMD: 2.30%; 95% CI: 1.40, 3.20; *P*<0.00001) (Figure 3). Significant heterogeneity was found (heterogeneity *I* ^2^ = 67%, *P* = 0.0007). Meta-regression indicated that health status might also be the main effect modifier (*P*<0.05). The dose of grape polyphenols, the intervention with alcohol or without alcohol, the baseline FMD level and the average age of the participants were not effect modifiers.

Subgroup analysis showed that the grape polyphenols could significantly increase FMD level 60 min after the intervention in the healthy subgroup (8 comparisons; WMD: 1.73%; 95% CI: 0.84, 2.62; *P* = 0.0001). The percentage change in FMD in the subgroup with high cardiovascular risk factors 60 min after intervention (2 trials; WMD: 3.61%; 95% CI: 1.78, 5.44; *P* = 0.0001) was much higher than in the healthy subgroup. Furthermore, there was no significant heterogeneity in the healthy subgroup (heterogeneity *I* ^2^ = 49%, *P* = 0.06) (Figure 3, Table S4).

### Acute Effects of Grape Polyphenols on FMD 120 min after the Intervention

**Figure 4:**
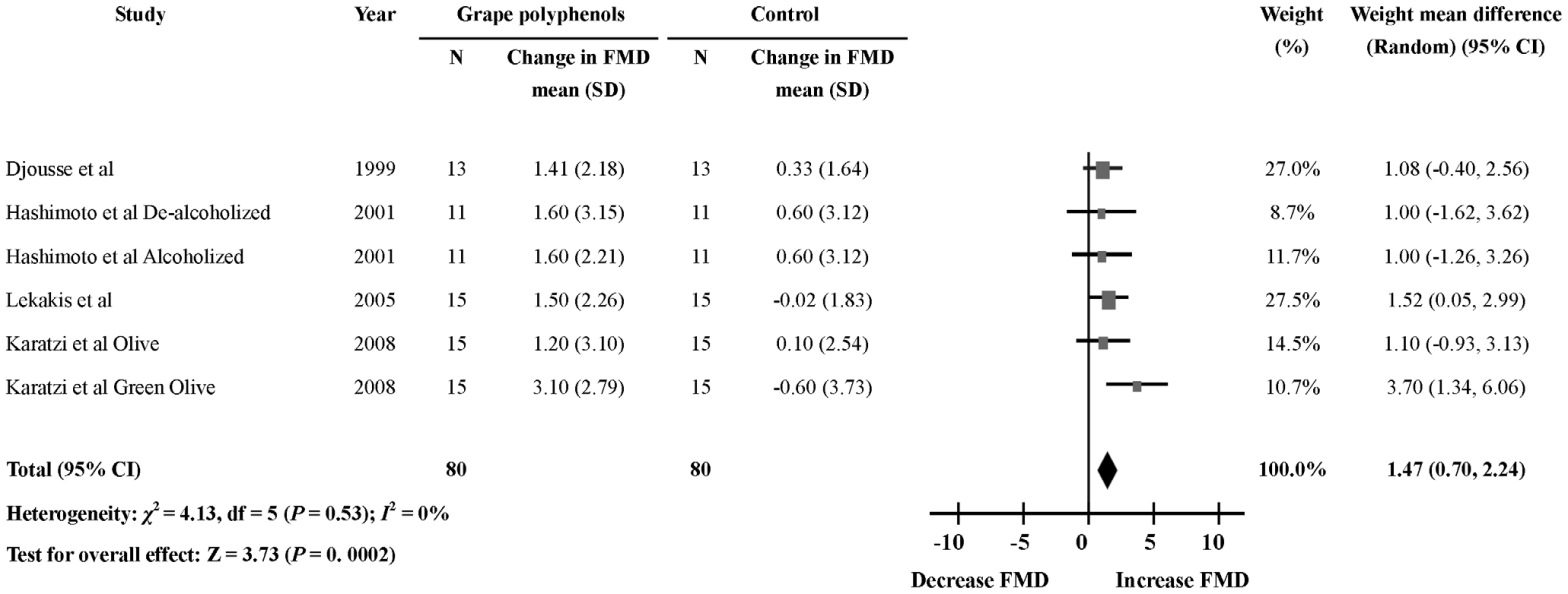
Meta-analysis of the 120 min effect of grape polyphenols on flow-mediated dilation (FMD) compared with controls. The sizes of the data markers indicate the weight of each study in the analysis. The subgroups were differentiated by health status of the subjects.

Four studies [12], [13], [15], [19] reported the effects of grape polyphenols on FMD 120 min after the ingestion. The results showed that there was significant change in FMD level 120 min after the ingestion of grape polyphenols (6 comparisons; WMD: 1.47%; 95% CI: 0.70, 2.24; *P* = 0.0002) (Figure 4). No significant heterogeneity was found in this meta-analysis (heterogeneity *I* ^2^ = 0%, *P* = 0.53).

### Acute Effects of Grape Polyphenols on FMD 180 min after the Intervention

**Figure 5:**
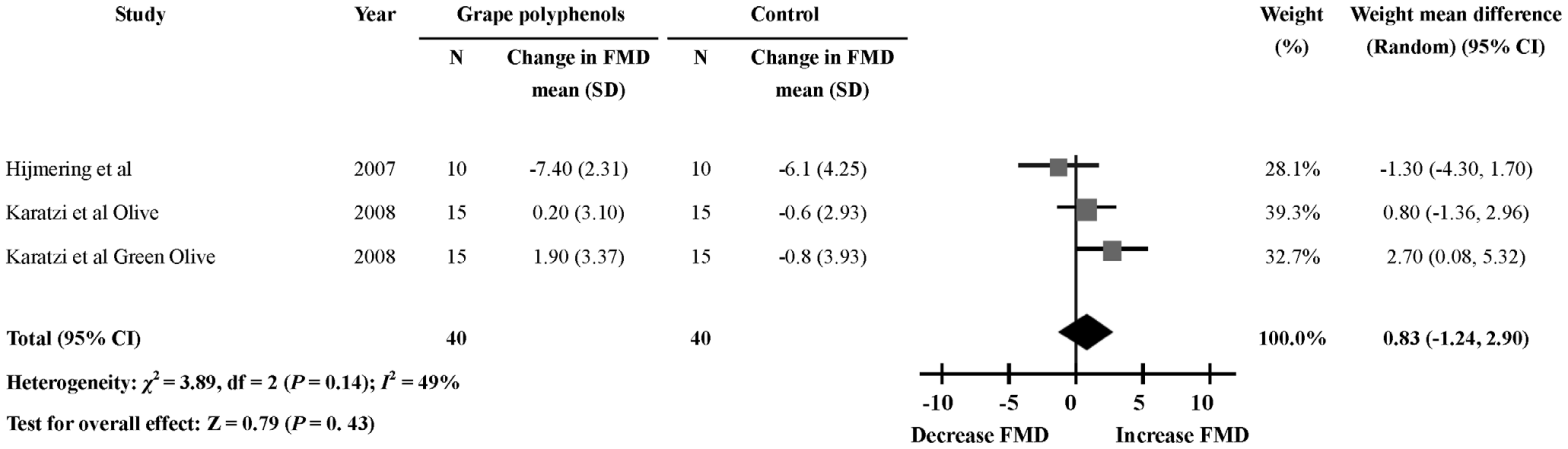
Meta-analysis of the 180 min effect of grape polyphenols on flow-mediated dilation (FMD) compared with controls. The sizes of the data markers indicate the weight of each study in the analysis. The subgroups were differentiated by health status of the subjects.

Only two studies [17], [19] reported on the effects of grape polyphenols on FMD 180 min after the ingestion. The results revealed no significant change of FMD level 180 min after the ingestion of grape polyphenols (3 comparisons; WMD: 0.83%; 95% CI: −1.24, 2.90; *P* = 0.43) (Figure 5). Significant heterogeneity was not detected in this meta-analysis (heterogeneity *I* ^2^ = 49%, *P* = 0.14).

### Publication Bias

Statistical analyses of the Egger test and funnel plots were performed to detect the publication bias. No publication bias was found in the three meta-analyses (Egger test, the meta-analysis 30 min after the intervention: *P* = 0.651; the meta-analysis 60 min after the intervention: *P* = 0.470; the meta-analysis 120 min after the intervention: *P* = 0.605; the meta-analysis 180 min after the intervention: *P* = 0.770).

## Discussion

The present meta-analyses indicate that the FMD level was significantly increased in the initial two hours after intake of grape polyphenols in adults when compared to control subjects. Significant heterogeneity was detected in the 30 min and 60 min meta-analyses, and meta-regression and subgroup analyses were performed and found that health status was the main effect modifier. Therefore, subgroups based on health status were introduced and the results revealed that intake of grape polyphenols could significantly increase FMD level in healthy adults in the initial two hours after the intervention, and the peak effect of grape polyphenols on FMD was found to be at 30 min after ingestion. Differently, the FMD level appeared to be much more obviously increased in subjects with significant cardiovascular risk factors when compared to healthy subjects in the initial one hour, and the peak effect of grape polyphenols on FMD was found 60 min after ingestion. Furthermore, the heterogeneity could be largely explained by the health status, and no significant heterogeneity was detected in the healthy subgroups. Potentially, the acute effects of grape polyphenols on endothelial function appear to be more significant but delayed in subjects with high cardiovascular risk factors than in healthy subjects.

*In vitro* studies have shown significant increases in nitric oxide in human umbilical vein endothelial cells treated with grape polyphenol extract [7], [47]. Hooper *et al.* [4] conducted a meta-analysis in 2007 to investigate the acute effects of grape polyphenols on endothelial function in humans. However, Hooper only included four trials in his meta-analysis and the acute effects of grape polyphenols on endothelial function were not explored at fixed times (e.g., 30 min, 60 min, 120 min after ingestion). Many new trials investigating the acute effects of grape polyphenols on endothelial function have been performed since 2007 [17]–[20]. Therefore, new meta-analyses are warranted to clarify these acute effects. The present meta-analyses revealed that intake of grape polyphenols could significantly increase the FMD level in both healthy subjects and subjects with high cardiovascular risk factors, but the increased FMD level appeared to be much more obvious in subjects with high cardiovascular risk factors. Moreover, the peak effect of grape polyphenols on FMD in healthy subjects was found 30 min after ingestion, which was different from the effect in subjects with high cardiovascular risk factors (the peak effect was found 60 min after ingestion).

Goldberg *et al.* have detected the absorptive efficiency of grape polyphenols and showed that in healthy subjects the concentration of grape polyphenols peaked in serum around 30 min after oral ingestion [48]. Manach *et al.* have found that the maximum plasma antioxidant capacity was usually reached 1–4 h after the ingestion of polyphenols [49]. Our meta-analyses revealed that the FMD level was significantly increased in the initial two hours after oral ingestion of grape polyphenols, and the peak FMD level emerged 30 min after the intervention in healthy adults. These data were consistent with the findings of Goldberg and Manach. However, compared with healthy subjects, the FMD level after the initial first hour might be more significantly increased in subjects with high cardiovascular risk factors, but the peak effect was delayed until 60 min after the ingestion. The difference in acute effects on FMD between the two subgroups might be ascribed to the impaired endothelial cells in disease conditions. In the subgroup with high cardiovascular risk factors, the subjects were smokers or patients with coronary heart disease. Smoking has been demonstrated to inhibit the release of nitric oxide and damage the endothelial cells [50], which in turn have been considered an early feature of atherosclerosis [2], [3]. The data indicated that baseline FMD levels in the subgroup with high cardiovascular risk factors varied from 2.6% to 5.65%, which were evidently lower than in the healthy subgroup (ranged from 5.4% to 7.4%). This further supports the above results. Therefore, in these subjects with high cardiovascular risk factors, endothelial cells have been impaired and might require 60 min after the ingestion of grape polyphenols to produce adequate nitric oxide to improve endothelial function, resulting in the delayed effect of grape polyphenols on endothelial function. However, the exact mechanisms are still unclear and need to be explored in the future.

Despite the intriguing results of the present meta-analysis, some potential limitations should be addressed. First, the number of included trials and the sample size in each study are both relatively small, and some trials were not randomized, placebo-controlled studies. Hence, well-designed and larger trials are needed in the future to verify our present results.

Second, the sources of grape polyphenols in the included trials were not consistent. Seven studies used red wine for the intervention [12]–[14], [16]–[19], whereas red grape polyphenol extract [15] or organic red grape juice [20] were applied in the other two studies. Inconsistent sources might influence the acute effects of grape polyphenols on FMD. Future trials with consistent source of grape polyphenols are needed to be performed.

Third, the baseline cholesterol concentration in the present meta-analyses was less than 6.0 mmol/L in six included studies, but not reported in other three [13], [16], [20]. The baseline triglyceride concentration was only reported in three trials [12], [15], [17], and ranged from 1.0 mmol/L to 1.80 mmol/L. However, the changes of lipid levels were only reported in two studies [12], [17] duo to the supplementation of grape polyphenols, which were insufficient to conduct a meta-analysis. Therefore, more studies focusing on the effect of grape polyphenols on blood lipid should be performed in the future to clarify this issue.

Fourth, the levels of inflammatory markers (e.g., C-reactive protein), cytokines (e.g., Interleukin-6) and adipokines (e.g., Leptin) might be also influenced by the supplementation of grape polyphenols, because recent study has indicated grape polyphenols might improve the inflammatory status in patients with cardiovascular disease [51]. However, only Hijmering’s trial [17] reported the baseline and changes of C-reactive protein in the present meta-analyses, and the result showed that no significant change of C-reactive protein level could be found in healthy subjects [17]. Studies focusing on the changes of inflammatory markers, cytokines and adipokines are needed in the future, especially in subjects with high cardiovascular risk factors.

Fifth, although there were 7 trials investigating the chronic effect of grape polyphenols on endothelial function [32]–[38], only 3 trials could be used to conduct a meta-analysis to assess the chronic effect of grape polyphenols on the endothelial function [32], [33], [35]. Other 4 studies were excluded for the following reasons: the exact values of FMD were not reported in two trials [36], [38]; the baseline FMD was not measured in one trial [37]; all the groups designed in the study used grape polyphenols and no blank control group was included [34]. The results from the meta-analysis of insufficient trials (only 3 chronic studies) may indicate an uncertain conclusion, so more well-designed studies are required in the future to clarify the chronic effect of grape polyphenols on endothelial function.

In conclusion, the present meta-analyses reveal that endothelial function can be significantly improved in the initial two hours after intake of grape polyphenols in healthy adults. The acute effects of grape polyphenols on endothelial function may be more significant but the peak effect is delayed in subjects with a history of smoking or coronary heart disease as compared with healthy subjects. Additional well-designed trials, especially in patients with dyslipidemia or cardiovascular diseases, are required to verify our present results.

## Supporting Information

Table S1 **The protocol of the present meta-analyses.**
(DOC)

Table S2 **PRISMA 2009 checklist.**
(DOC)

Table S3 **Subgroup analyses for the 30 min effect of grape polyphenols on endothelial function.**
(DOC)

Table S4 **Subgroup analyses for the 60 min effect of grape polyphenols on endothelial function.**
(DOC)
