# Supplementary material for: The Acute Effects of Grape Polyphenols Supplementation on Endothelial Function in Adults: Meta-Analyses of Controlled Trials
Source: PLoS One. 2013 Jul 24;8(7):e69818. doi: 10.1371/journal.pone.0069818 (PMC3722169; doi:10.1371/journal.pone.0069818)
Supplement: Table S1 — The protocol of the present meta-analyses. (DOC) [file pone.0069818.s001.doc]

**Table S1 The protocol of the present meta-analyses**

***Review question(s)***

How do grape polyphenols impact on endothelial function in adults in acute stage?

***Searches***

We systematically searched PubMed, EMBASE, and the Cochrane Library for published reports by using the query " (grape) OR (polyphenol) OR (red wine)" paired with "(endothelial) OR (endothelium)".

Reference lists of articles were also analyzed using a manual approach. Studies were chosen for analysis if they met the following criteria:

(i) the article should be published in English;

(ii) the study should be controlled trial in adults;

(iii) the endothelial function should be evaluated by the flow-mediated vasodilation method;

(iv) FMD should be measured after the intake of the grape polyphenols;

(v) the values of FMD were reported at the start and end of the intervention.

***Types of study to be included***

We will identify all published and controlled trials of grape polyphenols to evaluate the acute and long-term effect of grape polyphenols on FMD in adults.

***Condition or domain being studied***

Endothelial dysfunction has been considered as an early feature in the progression of atherosclerosis and an independent predictor of poor prognosis in many cardiovascular diseases.

***Participants/ population***

Inclusion:

(1) the subjects should be adults;

(2) the endothelial function has been measured by flow-mediated vasodilation (FMD) in the subjects.

Exclusion:

(1) Adolescents (under 18 years of age);

(2) Subjects were excluded if they had a familial or personal history of psychiatric disorders, epilepsy and sleep disorders.

***Intervention(s), exposure(s)***

The grape polyphenols control interventions are performed in adults that were given food or drink containing grape polyphenol extract (intervention group) or similar food or drink without grape polyphenol extract (control group). The grape polyphenol extract was prepared from the red grape. After supplementation of grape polyphenol extract, endothelial functions in these subjects were measured by a noninvasive ultrasound method (Flow-mediated vasodilation method) at fixed time (e.g. 30min, 60min, 2h, etc.) after the supplementation. Flow-mediated vasodilation (FMD) of the brachial artery is a noninvasive ultrasound method to assess endothelial function, which has been carried out by most of the trials investigating the endothelial function in adults.

***Comparator(s)/ control***

A non-exposed control group

Control group should be given similar drinks or food to the intervention group but without grape polyphenol extract.

***Outcomes***

Primary outcomes

The primary outcome was the percentage change in FMD between baseline and final levels due to grape polyphenols supplementation.

***Data extraction, (selection and coding)***

The search, data extraction, and quality assessment will be completed independently by two reviewers according to the inclusion criteria. Any discrepancies between the two reviewers will be resolved through discussion until a consensus is reached. The extracted data include the study characteristics (authors, publication year, sample size, study design, study duration, source and dose of grape polyphenols), population information (age, healthy status, baseline cholesterol concentration), and the values of baseline and final FMD.

***Risk of bias (quality) assessment***

The risk of bias in included studies will be assessed by two review authors considering the following characteristics:

Treatment allocation concealment: was the allocated treatment adequately concealed from study participants and clinicians and other healthcare or research staff at the enrolment stage?

Blinding: were the personnel assessing outcomes and analyzing data sufficiently blinded to the intervention allocation throughout the trial?

Completeness of outcome data: were participant exclusions, attrition and incomplete outcome data adequately addressed in the published report?

Selective outcome reporting: is there evidence of selective outcome reporting and might this have affected the study results?

Publication bias: Publication bias will be assessed with the Egger regression test and funnel plots.

Disagreements between the review authors over the risk of bias in particular studies will be resolved by discussion, with involvement of a third review author where necessary.

***Strategy for data synthesis***

We will provide a narrative synthesis of the findings from the included studies, structured around the type of intervention (controlled trial), target population characteristics, type of outcome (the percentage change in FMD between baseline and final levels due to grape polyphenols supplementation) and intervention content. We will provide summaries of intervention effects for each study by calculating standardized mean differences.

We anticipate that meta-analysis can be performed because of the outcome measured across the small number of existing trials. However, where studies have used the same type of intervention and comparator, with the same outcome measure, we will pool the results using random-effects model for meta-analyses, with standardized mean differences for FMD level. Weighted mean differences and 95% confidence intervals (CIs) will be calculated for net changes in FMD values. Statistical heterogeneity of treatment effects between studies will be formally tested with Cochrane’s test (*P* < 0. 1). The *I*^2^ statistic will also be examined, and we will consider an *I*^2^ value > 50% to indicate significant heterogeneity between the trials. Potential heterogeneity in estimates of treatment effect will be explored by univariate meta-regression or subgroup analyses. Publication bias will also be assessed with the Egger regression test and funnel plots.

***Analysis of subgroups or subsets***

If the necessary data are available, subgroup analyses will be done for people with healthy status separately.
