# Supplementary material for: The Acute Effects of Grape Polyphenols Supplementation on Endothelial Function in Adults: Meta-Analyses of Controlled Trials
Source: PLoS One. 2013 Jul 24;8(7):e69818. doi: 10.1371/journal.pone.0069818 (PMC3722169; doi:10.1371/journal.pone.0069818)
Supplement: Table S3 — Subgroup analyses for the 30 min effect of grape polyphenols on endothelial function. (DOC) [file pone.0069818.s003.doc]

**Table S3 Subgroup analyses for the 30 min effect of grape polyphenols on endothelial function**

|  | Intervention group | Effect (95% CI) | *P* |
| --- | --- | --- | --- |
| Age |  |  |  |
| < 29, low median | 4 | 2.40 (-0.20, 4.99) | 0.152 |
| ≥ 29, high median | 5 | 2.83 (2.01, 3.65) |  |
| Dose of grape polyphenols |  |  |  |
| < 650mg, low median | 5 | 2.59 (0.61, 4.57) | 0.987 |
| ≥ 650mg, high median | 4 | 2.60 (1.32, 3.88) |  |
| Alcohol or not |  |  |  |
| With alcohol | 4 | 2.15 (1.28, 5.37) | 0.067 |
| Without alcohol | 5 | 3.29 (1.42, 5.07) |  |
| Health status |  |  |  |
| Healthy | 6 | 2.55 (1.28, 3.82) | 0.046 |
| high cardiovascular risk factors | 3 | 2.90 (0.71, 5.09) |  |
| Baseline FMD level |  |  |  |
| < 5.5%, low median | 5 | 2.55 (1.25, 3.85) | 0.277 |
| ≥ 5.5%, high median | 4 | 2.64 (0.62, 4.65) |  |

FMD, flow-mediated dilation.
