# Supplementary material for: The Acute Effects of Grape Polyphenols Supplementation on Endothelial Function in Adults: Meta-Analyses of Controlled Trials
Source: PLoS One. 2013 Jul 24;8(7):e69818. doi: 10.1371/journal.pone.0069818 (PMC3722169; doi:10.1371/journal.pone.0069818)
Supplement: Table S4 — Subgroup analyses for the 60 min effect of grape polyphenols on endothelial function. (DOC) [file pone.0069818.s004.doc]

**Table S4 Subgroup analyses for the 60 min effect of grape polyphenols on endothelial function**

|  | Intervention group | Effect (95% CI) | *P* |
| --- | --- | --- | --- |
| Age |  |  |  |
| < 29.5, low median | 6 | 2.50 (1.27, 3.73) | 0.301 |
| ≥ 29.5, high median | 5 | 2.04 (0.55, 3.52) |  |
| Dose of grape polyphenols |  |  |  |
| ≤ 650mg, low median | 5 | 2.91 (1.69, 4.14) | 0.117 |
| > 650mg, high median | 6 | 1.71 (0.48, 2.93) |  |
| Alcohol or not |  |  |  |
| With alcohol | 6 | 2.61 (1.27, 3.94) | 0.152 |
| Without alcohol | 5 | 1.97 (0.66, 3.28) |  |
| Health status |  |  |  |
| Healthy | 8 | 1.73 (0.84, 2.62) | 0.012 |
| high cardiovascular risk factors | 3 | 3.61 (1.78, 5.44) |  |
| Baseline FMD level |  |  |  |
| < 5.7%, low median | 5 | 2.63 (1.27, 4.00) | 0.139 |
| ≥ 5.7%, high median | 6 | 1.97 (0.69, 3.25) |  |

FMD, flow-mediated dilation.
